# TripSense: A Trust-Based Vehicular Platoon Crowdsensing Scheme with Privacy Preservation in VANETs

**DOI:** 10.3390/s16060803

**Published:** 2016-06-01

**Authors:** Hao Hu, Rongxing Lu, Cheng Huang, Zonghua Zhang

**Affiliations:** 1 School of Electrical and Electronic Engineering, Nanyang Technological University, Singapore 639798, Singapore; hhu002@e.ntu.edu.sg (H.H.); huangcheng@ntu.edu.sg (C.H.); 2 IMT/TELECOM Lille, CNRS UMR 5157 SAMOVAR Lab, Lille 59650, France; zonghua.zhang@telecom-lille.fr

**Keywords:** trust, privacy, platoon, crowdsensing, Vehicular Ad Hoc Networks

## Abstract

In this paper, we propose a trust-based vehicular platoon crowdsensing scheme, named TripSense, in VANET. The proposed TripSense scheme introduces a trust-based system to evaluate vehicles’ sensing abilities and then selects the more capable vehicles in order to improve sensing results accuracy. In addition, the sensing tasks are accomplished by platoon member vehicles and preprocessed by platoon head vehicles before the data are uploaded to server. Hence, it is less time-consuming and more efficient compared with the way where the data are submitted by individual platoon member vehicles. Hence it is more suitable in ephemeral networks like VANET. Moreover, our proposed TripSense scheme integrates unlinkable pseudo-ID techniques to achieve PM vehicle identity privacy, and employs a privacy-preserving sensing vehicle selection scheme without involving the PM vehicle’s trust score to keep its location privacy. Detailed security analysis shows that our proposed TripSense scheme not only achieves desirable privacy requirements but also resists against attacks launched by adversaries. In addition, extensive simulations are conducted to show the correctness and effectiveness of our proposed scheme.

## 1. Introduction

Envisioned as one of the most promising applications to implement intelligent transportation systems (ITS), vehicular platooning [1,2] has the potential to enhance road safety, improve traffic efficiency and reduce energy consumption due to air drag reduction [3]. At the same time, with the increasing popularity of mobile devices and sensing technologies, a new sensing paradigm, mobile crowdsensing, attracts attention from both academia and industry [4]. Different from traditional sensor networks, this new sensing paradigm leverages the power of crowds for large scale sensing tasks and fuels the evolution of the Internet of Things (IoT) [5]. Many factories are built in remote areas where the sensor resources are limited, if the authority needs to inspect those factories, it can hardly collect information with the existing traditional sensor networks. However, given the fact that many highways go through the remote areas. One solution to this problem is to invite vehicles passing by those areas to take part in the crowdsensing tasks and utilize their sensed data (e.g., temperature, humidity, noise level, air pollution level, *etc.*).

However, due to the inherent openness of this platform, it is easy for vehicles to contribute corrupted data [6]. As a result, several research efforts have been made on ensuring the trustworthiness of the sensed data [7,8]. One possible solution is to establish the reputation system for evaluating the trustworthiness of volunteer contributions in participatory sensing applications [6].

**Figure 1:**
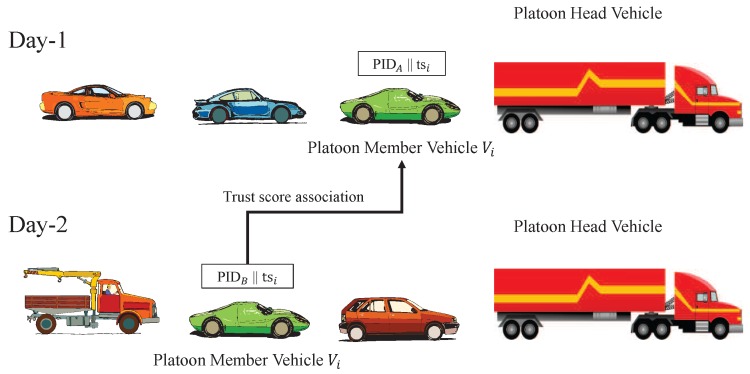
Platoon head vehicle associates a platoon member vehicle’s trust scores even when its pseudonym has been changed.

Furthermore, this new data aggregation way may also bring in privacy concerns into the networks. For example, the sensed data could reveal the capacity of a vehicle’s sensor and hence reveal the personal information of the vehicle. Another factor that has always been a concern is the location privacy, since the locations of the vehicles are closely related to the drivers of those vehicles [9]. To achieve location privacy, one approach is to use unlinkable pseudonyms that are periodically changed when broadcasting messages [10,11,12]. However, pseudonyms do not always ensure privacy, as an example shown in Figure 1, a platoon head vehicle is asking its platoon member vehicle $V_{i}$ for participating in the sensing task. When $V_{i}$ responds by sending its own reputation score $ts_{i}$ at Day-1 and Day-2, respectively, the platoon head vehicle can still associate $V_{i}$ in different days by associating its trust scores even when its pseudonym has been changed. The unchanged trust score of a vehicle reveals its location privacy and the platoon head vehicle can even derive the driving pattern of the platoon member vehicle. Therefore, it is compelling for us to build a trust system through which vehicles take its advantages without sacrificing their privacies, and a data aggregation mechanism to ensure data privacy.

Based on the observations above, we propose a trust-based vehicular platoon crowdsensing scheme, called TripSense, to improve sensing accuracy while achieving location and data privacy. This scheme is based on vehicular platooning technique to collect and aggregate data. At the same time, by establishing a trust model to measure the accuracy of a vehicle’s sensed data, the service provider (SP) efficiently detects and then excludes the malicious or selfish vehicles who submit corrupted sensed data. Meanwhile, the proposed scheme is characterized by its ability to preserve the location privacy and data privacy of sensing vehicles. With the assistance of platoon head vehicles, the communication overhead and computational cost can be greatly reduced. Specifically, our work features the following:

- First, we establish the trust system based on Dirichlet distribution to evaluate the sensing accuracy of all sensing vehicles in our proposed TripSense scheme. The historical sensed data will be evaluated and finally form a reputation score. Therefore, the sensing accuracy will be improved greatly when the data are always collected from those high reputation sensing vehicles.
- Second, we propose the TripSense scheme by taking advantages of the unique features of vehicular platooning. In this scheme, platoon head vehicles firstly authenticate all sensing vehicles inside the platoon and then select some of them according to their trust values. Later, the sensed data from sensing vehicles will be collected and aggregated by platoon head vehicles before they are finally uploaded to the server. Compared with previous works, our proposed scheme reduces the communication overhead and hence is more suitable for the dynamic and ephemeral vehicular *ad hoc* network.
- Third, we design a privacy-preserving sensing vehicle selection scheme based on our trust system and a privacy-preserving data aggregation scheme based on the efficient commitment scheme in [13] such that platoon head vehicles can collect the data without leaking sensing vehicles’ privacy.

The remainder of this paper is organized as follows. In Section 2, we formalize the system model, trust model and threat model considered in our work, and identify our design goals. In Section 3, we briefly recall the bilinear pairing and the Dirichlet distribution which have been applied in the trust and reputation system. In Section 4, the TripSense scheme is presented in detail, together with the rationale on how it can help the requesting vehicles choose a highly reliable relay vehicle without knowing its reputation score. Security analysis is then presented in Section 5, and the performance analysis is given in Section 6. Finally, we present the related work in Section 7 and draw conclusions in Section 8.

## 2. Problem Statement

In this section, we define the problem by formalizing the system model, security model and design goal.

### 2.1. System Model

**Figure 2:**
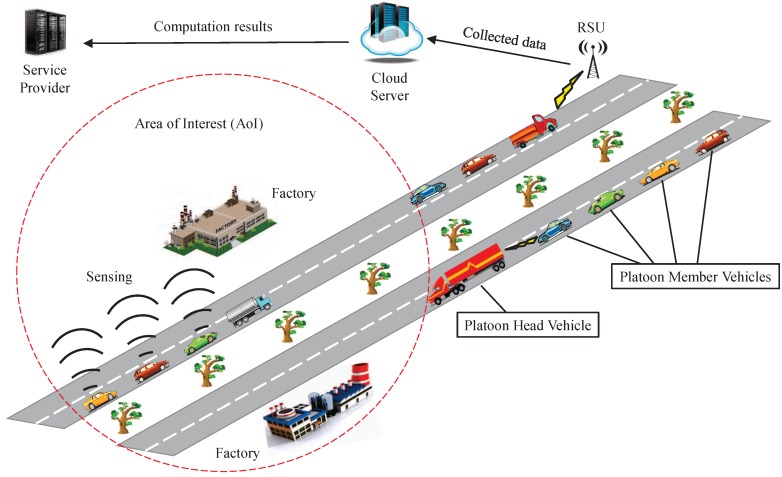
System model under consideration.

In our model, the service provider (SP) wants to inspect an area of interest (AoI) located near a highway where many platoons pass by. As illustrated in Figure 2, our system model consists of three roles: the service provider (SP), a cloud server (CS), the immobile roadside units (RSUs) along the highway and mobile vehicles traveling on the highway, which are equipped with onboard units (OBUs) and powerful sensors.

Service Provider (SP): The SP is fully trusted because it is normally controlled by the authority who wants to inspect an area of interest by collecting the data of this AoI. The data collected is a vector of readings regarding, for example, air pollution level, noise level, temperature, humidity and so on. The duty of SP is to initialize the whole system, and distribute key materials to RSUs and vehicles. It is also responsible for storing and updating trust values for all vehicles.

Roadside Units (RSUs): The RSUs are subordinated by the SP, which are connected to the CS and SP via reliable communication channels. Equipped with wireless devices, RSUs are able to exchange data with the vehicles passing-by. However, due to the high cost of RSU installment and maintenance, especially in the early stage of VANET, RSUs are sparsely deployed along the highway. The RSUs will never disclose any internal information without permission. However, we do not rule out the possibility that a portion of RSUs at the road side are compromised or the attackers even deploy bogus RSUs. Nevertheless, the SP can inspect all RSUs at high level: once the RSUs are compromised, they will be recovered or revoked soon by SP.

Cloud Server (CS): A CS collects data from RSUs, then aggregates them in a privacy-preserving way. In addition, a CS also computes the sensed data evaluations for vehicles and returns the results to SP. A CS is assumed to be honest but curious about the sensed data of vehicles, which means that it follows the proposed scheme faithfully but tends to be curious and disclose vehicles’ privacy.

Vehicles: The vehicles are regarded as a group of highly mobile nodes equipped with OBUs which allow them to communicate with other vehicles or with RSUs. On the highway, vehicles follow platoon head vehicles to form a platoon. With this driving pattern, the vehicles can be further divided into two categories:

- Platoon Head (PH) Vehicles $P_{0}=\left\{ph_{1},ph_{2},\cdots \right\}$: PH vehicles take full control of the whole platoon when driving on the highway, and they are responsible for the safety and user experience of all platoon member vehicles. Apart from that, they also claim a sensing task and submit sensed data to RSUs through V-2-I communication. PH vehicles are also honest but curious about the privacy of platoon member vehicles. In fact, PH vehicles could be malicious and provide untruthful aggregated data to server in order to subvert the system, or they may even collude with a bunch of PM vehicles with the objective to victimize other PM vehicles. However, in this work, we do not consider this issue since it is not the main focus of this work.
- Platoon Member (PM) Vehicles $V_{0}=\left\{v_{1},v_{2},\cdots \right\}$: Each PM vehicle is equipped with various types of powerful sensors to meet the requirements of different tasks. Through V-2-V communication, a member vehicle authenticates itself and then submits its sensed data to the PH vehicles. Some of the PM vehicles are compromised by adversaries launch attacks, while other PM vehicles are all honest but curious.

Both PH vehicles and PM vehicles will be get paid by the SP for leading a platoon or contributing their sensed data.

### 2.2. Security Model

In our security model, we assume that all roles, except the SP, RSUs and malicious PM vehicles, are honest-but-curious, *i.e.*, they will faithfully follow the protocol, but could also snoop into another role’s privacy on account of some sensitive information available to them. Specifically, we first consider the privacy requirements of PM vehicles.

Privacy requirements of platoon member vehicles: The privacy requirements of a PM vehicle include its data privacy, location privacy and identity privacy. Since the sensed data are private assets of a PM vehicle, which may reflect some sensitive information like the sensor accuracy or sensing ability, the PM vehicles will not disclose them to others. The location privacy requirement indicates that a PM vehicle will not let its PH vehicle know its past driving pattern, and the identity privacy means that the PM vehicle tries to keep his real identity secret. Meanwhile, each vehicle is also privacy-curious, *i.e.*, it tends to disclose the privacy of other vehicles from other information available to it.

We assume that there are two kinds of adversaries according to their attacking abilities, the first kind tries to impersonate another authorized PM vehicle; the second kind is able to control a small portion of vehicles. Specifically, we list potential attacks as follows:

- *Impersonation attack:* The first kind of adversary may try to impersonate a PM vehicle to ask for a sensing task. However, this PM vehicle may not be qualified in the system. Once chosen to fulfill the task, these unqualified vehicles may submit inaccurate sensed data.
- *Malicious sensing attack:* The second kind of more capable adversary is able to control a small fraction of the vehicles in the system who submit inaccurate sensed data deliberately to subvert the system. Another possible case is that the PM vehicle is selfish, so it reports arbitrary data without using sensors to save power.
- *Trust score association attack:* PH vehicles are honest but curious, in this case, if the trust score of a PM is directly given to its PH vehicle, its driving pattern will be disclosed. As described in Section 1, the reason is because every time the PH vehicle can associate a PM vehicle in platoon according to the same trust score collected in different trips, even though the pseudo-id has been changed.
- *Data analysis attack:* Due to the curious characteristics of both PH vehicles and PM vehicles, they may eavesdrop on the transmission of sensed data and try to analyze the data. On the other hand, a cloud server (CS) is also curious about the sensed data. If the data is not encrypted, these attackers can easily analyze the data in transmission.

### 2.3. Design Goal

Our design goal is to develop a trust-based privacy-preserving scheme to not only improve the sensing accuracy, but also preserve the privacy of sensing vehicles while resisting against the attacks launched by adversary. Specifically, the following desirable objectives need to be achieved.

- *Ensuring the sensed data reliability and accuracy.* According to our adversary model, the existence of selfish and malicious vehicles who submit corrupted sensed data will make the final results inaccurate and unreliable. Hence, our proposed scheme should be able to improve the sensed data accuracy by excluding selfish and malicious vehicles’ data.
- *Achieving privacy-preserving sensing vehicle selection, sensed data aggregation and evaluation.* The proposed scheme should achieve privacy requirements of PM vehicles. Particularly, (i) the real identity of PM vehicles will never be disclosed; (ii) when a PM vehicle replies to the PH vehicle, the PH vehicle can never know the exact trust score of this PM vehicle; (iii) when a PH vehicle collects and aggregates the sensed data, it can never know what the data is; (iv) the CS can never know the aggregated sensed data and the evaluations on that data.
- *Resisting against attacks launched by adversaries.* The proposed scheme should also be secure and reliable in VANET. Once an outside adversary launches some attacks, e.g., impersonation attack or data analysis attack, the proposed scheme should be able to detect them.

## 3. Preliminaries

### 3.1. Bilinear Pairing

Let G and $G_{T}$ be two multiplicative cyclic groups of the same composite order *n*. Then, a bilinear pairing $e:G\times G\to G_{T}$ will satisfy the following properties: (i) Bilinear: Let g,h∈G and $a,b\in Z_{n}^{*}$, then $e\left(g^{a},h^{b}\right)=e{\left(g,h\right)}^{ab}$; (ii) Non-degenerated: Let g∈G be a generator in G, then $e\left(g,g\right)\neq 1_{G_{T}}$; and (iii) Computable: Let g,h∈G, then e(g,h) can be efficiently computed.

**Definition 1**  (Bilinear Parameter Generator). *A bilinear parameter generator Gen is a probabilistic algorithm that takes a security parameter κ as its input, and outputs a six-tuple $\left(p,q,g,G,G_{T},e\right)$, where p,q are κ-bit prime numbers, n=p·q, $\left(G,G_{T}\right)$ are two multiplicative groups of the same order n, g∈G is a generator, and $e:G\times G\to G_{T}$ is a non-degenerated and efficiently computable bilinear map.*

### 3.2. Beta Distribution

Defined on the interval of [0,1], beta distribution is a family of continuous probability distributions indexed by two parameters *α* and *β*. A random variable X beta-distributed with parameters *α* and *β* can be denoted by: X∼Beta(α,β). Given that Gamma function is an extension of the factorial function where $\Gamma \left(\alpha \right)=\int_{0}^{\infty }x^{\alpha -1}e^{-x}dx$. The probability density function (PDF) f(x|α,β) can be expressed by using gamma function Γ as: $f\left(x|\alpha ,\beta \right)=\frac{\Gamma (\alpha +\beta )}{\Gamma (\alpha )\Gamma (\beta )}x^{\alpha -1}{\left(1-x\right)}^{\beta -1}$, where 0≤x≤1, α>0, β>0. The probability expectation value of the beta distribution is given by: $E\left(x\right)=\frac{\alpha }{\alpha +\beta }$.

**Figure 3:**
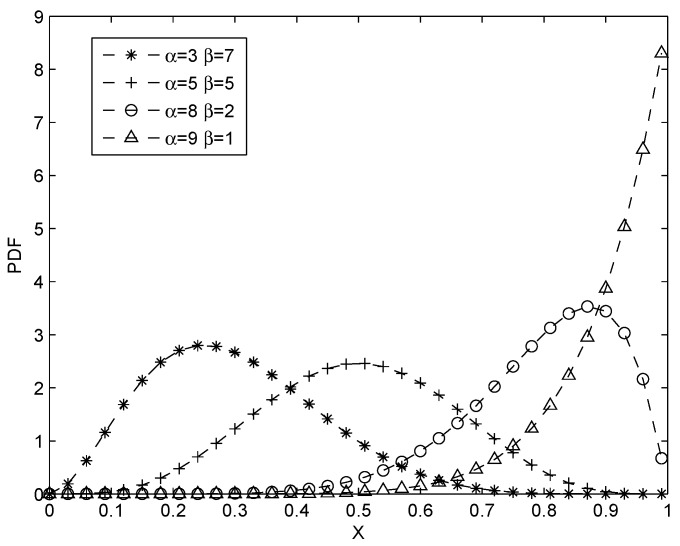
PDF of beta distribution with parameter *α* and *β*.

Figure 3 shows the PDF of beta distribution with different parameters *α* and *β*. It expresses the uncertain probability that a process will produce positive outcomes in future. Take the example, when α=8, β=2, according to expectation equation, the probability expectation value of this type of beta distribution is E(x)=0.8, which can be interpreted as the relative frequency of positive outcome that is somewhat uncertain and that the most likely value is 0.8.

### 3.3. Dirichlet Distribution

The Dirichlet distribution is a family of continuous multivariate probability distributions parameterized by *a priori* parameter vector $\vec{\alpha }$. It is the conjugate prior distribution for the parameters of the multinomial distribution. In the case of a binary state space, it is determined by the Beta distribution [14]. Generally, we can use the Dirichlet distribution to describe the probability distribution over a *k*-component random variable $\vec{X}=\left\{X_{1},X_{2},\cdots ,X_{k}\right\}$. If $\vec{p}=\left\{p_{1},p_{2},\cdots ,p_{k}\right\}$ is the probability distribution vector of *X*, and it satisfies $P\left\{\theta_{i-1}<X_{i}\leq \theta_{i}\right\}=p_{i}$ $\left(1\leq i\leq \;k,\theta_{i}\in \left[0,1\right],\theta_{i+1}>\theta_{i}\right)$. The Dirichlet distribution captures a sequence of observations of *k* possible outcomes, and those observations serve as the prior parameters $\vec{\alpha }=\left(\alpha_{1},\alpha_{2},\ldots ,\alpha_{k}\right)$ that denote the cumulative observations and initial beliefs of *X*. $\vec{p}$ is a *k*-dimensional random variable and $\vec{\alpha }$ is a *k*-dimensional random observation variable. The probability density function is given by:

(1) $$f\left(\vec{p}|\vec{\alpha }\right)=\frac{\Gamma (\Sigma_{i=1}^{k}\alpha_{i})}{\prod_{i=1}^{k}\Gamma \left(\alpha_{i}\right)}\prod_{i=1}^{k}p_{i}^{\alpha_{i}-1}$$

where $0\leq p_{1},p_{2},\cdots ,p_{k}\leq 1$; $\sum_{i=1}^{k}p_{i}=1$; $\alpha_{1},\alpha_{2},\ldots ,\alpha_{k}>0$. The expected value of the probability that *X* to be $x_{i}$ given the observations vector $\vec{\alpha }$ is given by: $E\left(p_{i}|\vec{\alpha }\right)=\frac{\alpha_{i}}{\sum_{i=1}^{k}\alpha_{i}}$. Furthermore, if we let $\alpha_{0}=\sum_{i=1}^{k}\alpha_{i}$, the variance of the event of *X* to be $x_{i}$ is given by: $Var\left[X=x_{i}\right]=\frac{\alpha_{i}\left(\alpha_{0}-\alpha_{i}\right)}{\alpha_{0}^{2}\left(\alpha_{0}+1\right)}$. If i≠j, the covariance is: $Cov\left[X=x_{i},X=x_{j}\right]=\frac{-\alpha_{i}\alpha_{j}}{\alpha_{0}^{2}\left(\alpha_{0}+1\right)}$.

## 4. Proposed TripSense Scheme

In this section, we propose our TripSense scheme, which consists of six parts: system initialization, trust-based privacy-preserving sensing vehicle selection, privacy-preserving sensed data aggregation, aggregated sensed data retrieval, privacy-preserving sensed data accuracy evaluation, and Dirichlet-based trust management.

### 4.1. System Initialization

We assume that a service provider (SP) will bootstrap the whole system. Specifically, given a security parameter *κ*, SP first generates the bilinear parameters $\left(p,q,g,G,G_{T},e\right)$ by running Gen(κ) and then computes $h=g^{q}\in G$. Next, SP chooses a secure symmetric encryption algorithm Enc(), *i.e.*, AES, and a collision-resistant cryptographic hash function $H:{\left\{0,1\right\}}^{*}\to Z_{n}^{*}$. In addition, SP chooses a random number $s\in Z_{n}^{*}$ as the master key and computes $P_{pub}=g^{s}$, n=pq. Finally, SP keeps p,q secret and publishes $\left\{n,g,h,P_{pub},G,G_{T},e,H,Enc\right\}$.

RSU REGISTRATION: For each RSU, SP first generates an identity, denoted by RID, and then calculates its private key and public key as ($s_{r}$; $S_{r}$), where $s_{r}$ is randomly chosen in $Z_{n}^{*}$ and $S_{r}=g^{s_{r}}$.

PH VEHICLE REGISTRATION: For any platoon head (PH) vehicle $ph_{i}\in P_{0}$ that wants to participate in the sensing task, it has to register itself to SP and obtain a real identity $RID_{i}$. Then, SP assigns the private key and public key to $ph_{i}$ as ($s_{i}$, $S_{i}$), where $s_{i}$ is randomly chosen in $Z_{n}^{*}$ and $S_{i}=g^{s_{i}}$.

PM VEHICLE REGISTRATION: For each platoon member (PM) vehicle $v_{j}\in V_{0}$ that wants to take part in the sensing task and contribute its sensed data, it first registers itself in the system. The following steps between SP and $v_{j}$ show the registration process.

- SP first chooses a random number $k_{0}\in Z_{n}^{*}$ and uses Enc() to compute pseudo-IDs ${\mathrm{PID}}_{j}=\left\{PID_{j1},PID_{j1},\cdots \right\}$, where each pseudo-ID $PID_{jk}\in {\mathrm{PID}}_{j}$ is computed as $PID_{jk}=Enc_{k_{0}}(ID_{j}\left|\right|r_{jk})$ with a fresh random number $r_{jk}\in Z_{n}^{*}$. Then, for each $PID_{jk}$, SP calculates its corresponding private key by $s_{jk}=H{\left(PID_{jk}\right)}^{s}$ and public key by $S_{jk}=g^{s_{jk}}$. Finally, SP sends ${\mathrm{PID}}_{j}$ and the corresponding public and private keys back to $v_{j}$ via a secure channel.
- After receiving pseudo-IDs ${\mathrm{PID}}_{j}$ and their private keys, $v_{j}$ verifies the correctness of each private key $s_{jk}$ by checking $e\left(H\left(PID_{jk}\right),P_{pub}\right)\overset{?}{=}e\left(s_{jk},g\right)$.

TRUST REGISTRATION: Each registered PM vehicle $v_{j}$ will be given a trust score $ts_{j}$ by SP before it is able to take part in the sensing task, where $ts_{j}\in \left[0,1\right]$ with the precision of two decimal places. Initially, $ts_{j}=ts_{0}$. In addition, SP also defines *L* trust levels $\left\{TL_{1},TL_{2},\cdots ,TL_{L}\right\}$ for all trust scores from 0 to 1. For instance, $TL_{1}$ is with (0,0.1], $TL_{2}$ is with (0.1,0.2], ⋯, $TL_{10}$ is with (0.9,1]. Later, SP selects *l* random elements $\left\{y_{1},y_{2},\cdots ,y_{L}\in Z_{n}^{*}\right\}$ as master keys, and publishes the public keys as $\left\{Y_{1}=g^{y_{1}},Y_{2}=g^{y_{2}},\cdots ,Y_{L}=g^{y_{l}}\right\}$.

- For a registered PM vehicle $v_{j}$ with trust score $ts_{j}\in TL_{x}$, where x∈[1,L], SP makes signatures for each of its pseudonyms $PID_{jk}\in {\mathrm{PID}}_{j}$ as $A_{jk}=g^{\frac{1}{y_{x}+ts_{j}+s_{jk}+H\left(T_{j}\right)}}$, where $T_{j}$ is the timestamp for updating the trust score of $v_{j}$.

TASK REGISTRATION: Before a task is broadcasted to PH vehicles, it should be registered by SP. First, the sensed data categories need to be decided, such as air pollution level, noise level, temperature, humidity, and so on. Second, the the format could be defined as $\vec{d}=\left(d^{A},d^{B},\cdots ,d^{Z}\right)$, where each element denotes one category. In addition, SP also defines that each piece of data has the precision of two decimal places. In addition, the location of AoI is also included in the task. Finally, the SP will also decide a sensing vehicle trust level threshold $TL_{TH}$ according to the accuracy requirements of the task to make sure that the sensed data only come from those more trusted vehicles.

### 4.2. Trust-Based Privacy-Preserving Sensing Vehicle Selection

We assume that there are a number of *m* PH vehicles in the system which would like to participate in the sensing task. They form a new set $P=\{ph_{1},ph_{2},\cdots ,ph_{m}\}$. For a specific PH vehicle $ph_{i}\in P$, it needs to collect sensed data from all registered PM vehicles in its platoon and then select those which meet the trust level requirement. Therefore, a trust-based privacy-preserving sensing vehicle selection scheme has been proposed as follows:

*Step 1:* When the platoon approaches the AoI, $ph_{i}$ broadcasts its sensing requests $\left\{PID_{i}||H{\left(T\right)}^{s_{i}}||T\right\}$ to all platoon members, where *T* is the current timestamp.

*Step 2:* In $ph_{i}$’s platoon, for each registered PM vehicle $v_{j}$, after receiving $ph_{i}$’s requests, it first verifies whether $ph_{i}$ is a registered PH vehicle by checking $e\left(H{\left(T\right)}^{s_{i}},g\right)\overset{?}{=}e\left(S_{i},H\left(T\right)\right)$. If it holds, $v_{j}$ accepts the requests, otherwise, $v_{j}$ rejects $ph_{i}$. Then $v_{j}$ responds to $ph_{i}$ with $\left\{PID_{jk}||H{\left(T^{\prime }\right)}^{s_{jk}}||TL_{x}||\Pi ||T^{\prime }||T_{j}\right\}$ by calculating as follows, where $T^{\prime }$ is the current timestamp and $T_{j}$ is the latest timestamp for updating $v_{j}$’s trust score.

- Since the trust scores of PM vehicles are two decimal places, we need to expand them by 100 times before they can be encrypted. If $v_{j}$’s trust score is with trust level $TL_{x}$ where x∈[1,L], $v_{j}$ encrypts the expanded trust score $ts_{j}$ as $C=g^{ts_{j}}h^{r}$, with a fresh random number $r\in Z_{n}^{*}$.
- Similarly, the sensed data are all collected and expanded by 100 times. For one category in $\vec{d}$, $v_{j}$ encrypts the expanded sensed data $d_{j}$ as $D=g^{d_{j}}h^{r^{\prime }}$, where $r^{\prime }$ is a random number in $Z_{n}^{*}$.
- $v_{j}$ chooses a pseudonym $PID_{jk}$ and a random element $v\in Z_{n}^{*}$ to calculate the following:
  (2) $$\begin{array}{cc} & B=A_{jk}^{v}=g^{\frac{v}{y_{x}+ts_{j}+s_{jk}+H\left(T_{j}\right)}}\\ & E=B^{-ts_{j}}\cdot g^{v}=g^{\frac{v(y_{x}+s_{jk}+H\left(T_{j}\right))}{y_{x}+ts_{j}+s_{jk}+H\left(T_{j}\right)}} \end{array}$$
- $v_{j}$ randomly chooses $ts_{j}^{\prime \prime }$, $r^{\prime \prime }$, $v^{\prime \prime }\in Z_{n}^{*}$ and computes $C^{\prime \prime }=g^{ts_{j}^{\prime \prime }}h^{r^{\prime \prime }}$, $E^{\prime \prime }=B^{-ts_{j}^{\prime \prime }}g^{v^{\prime \prime }}$.
- $v_{j}$ calculates the proofs $\Pi =\{C,B,E,D,z_{1},z_{2},z_{3},\phi \}$ as:
  (3) $$\begin{array}{cc} & \phi =H(C,B,E,D,C^{\prime \prime },E^{\prime \prime },H{\left(T\right)}^{s_{j}})\\ & z_{1}=ts_{j}^{\prime \prime }+\phi \cdot ts_{j}\;\;\;mod\;\;\;n\\ & z_{2}=r^{\prime \prime }+\phi \cdot r\;\;\;mod\;\;\;n\\ & z_{3}=v^{\prime \prime }+\phi \cdot v\;\;\;mod\;\;\;n \end{array}$$

*Step 3:* After receiving the response from $v_{j}$, $ph_{j}$ first checks whether $v_{j}$ is a registered PM vehicle by checking $e\left(g,H{\left(T^{\prime }\right)}^{s_{jk}}\right)\overset{?}{=}e\left(S_{jk},H\left(T^{\prime }\right)\right)$. Then, $ph_{i}$ checks whether the timestamp $T^{\prime }$ is relatively new. Next, $ph_{i}$ checks whether $v_{j}$’s trust score $ts_{j}$ is with $TL_{x}$ by checking $e\left(E,g\right)\overset{?}{=}e\left(B,Y_{x}\cdot S_{jk}\cdot g^{H(T_{j})}\right)$. If it does hold, $ph_{i}$ calculates $\hat{C}=g^{z_{1}}h^{z_{2}}C^{-\phi }$, $\hat{E}=B^{-z_{1}}g^{z_{3}}E^{-\phi }$ and checks $\phi \overset{?}{=}H\left(C,B,E,D,\hat{C},\hat{E},H{\left(T\right)}^{s_{i}}\right)$. If it holds, $ph_{i}$ will finally check whether $TL_{x}\geq TL_{TH}$, and accept $v_{j}$’s sensed data once its trust level satisfies the task requirement.

### 4.3. Privacy-Preserving Sensed Data Aggregation

For each PH vehicle $ph_{i}\in P$, where i∈[1,m], we assume that a number of $n_{i}$ PM vehicles meet the trust level threshold requirement, and those eligible PM vehicles form a set $V_{i}=\left\{v_{i1},v_{i2},\cdots ,v_{in_{i}}\right\}$. After a PH vehicle $ph_{i}$ receives the sensed data from its PM vehicles, it selects those eligible data and aggregates them locally before submitting to CS for global aggregation. Therefore, a privacy-preserving data aggregation scheme has been proposed.

Local Aggregation: Take one data category, $d^{A}$, in $\vec{d}$ as an example. For simplicity, we omit the superscript and use $D_{in_{i}}$ instead of $D_{in_{i}}^{A}$. As described in Section 4.2, $ph_{i}$ collects encrypted sensed data from $n_{i}$ PM vehicles as $D_{i1}=g^{d_{i1}}\cdot h^{r_{i1}^{\prime }},D_{i2}=g^{d_{i2}}\cdot h^{r_{i2}^{\prime }},\cdots ,D_{in_{i}}=g^{d_{in_{i}}}\cdot h^{r_{in_{i}}^{\prime }}$ together with their corresponding encrypted trust score: $C_{i1}=g^{ts_{i1}}\cdot h^{r_{i1}},C_{2}=g^{ts_{i2}}\cdot h^{r_{i2}},\cdots ,C_{in_{i}}=g^{ts_{in_{i}}}\cdot h^{r_{in_{i}}}$. Then, $ph_{i}$ aggregates the encrypted data $D_{ij}$ and trust score $C_{ij}$ of each PM vehicle $v_{ij}\in V_{i}$ where $j\in [1,n_{i}]$ using the paring:

(4) $$\begin{array}{cc} & e\left(D_{ij},C_{ij}\right)=e\left(g^{d_{ij}}\cdot h^{r_{ij}}\right)=e\left(g^{d_{ij}}\cdot h^{r_{ij}^{\prime }},g^{ts_{ij}}\cdot h^{r_{ij}}\right)\\ & =e{\left(g,g\right)}^{d_{ij}ts_{ij}}\cdot e{\left(g,h\right)}^{d_{ij}r_{ij}+ts_{ij}r_{ij}^{\prime }}\cdot e{\left(h,h\right)}^{r_{ij}r_{ij}^{\prime }} \end{array}$$

Later $ph_{i}$ aggregates all aggregated data of all PM vehicles in $V_{i}$ together as:

(5) $$\begin{array}{cc} & \phi_{i}=\prod_{j=1}^{m_{i}}e\left(D_{ij},C_{ij}\right)=\prod_{j=1}^{m_{i}}e\left(g^{d_{ij}}\cdot h^{r_{ij}}\right)\\ & =\prod_{j=1}^{m_{i}}e{\left(g,g\right)}^{d_{ij}ts_{ij}}\cdot \prod_{j=1}^{m_{i}}e{\left(g,h\right)}^{d_{ij}r_{ij}+ts_{ij}r_{ij}^{\prime }}\cdot \prod_{j=1}^{m_{i}}e{\left(h,h\right)}^{r_{ij}r_{ij}^{\prime }}\\ & =e{\left(g,g\right)}^{\sum_{j=1}^{m_{i}}d_{ij}ts_{ij}}\cdot e{\left(g,h\right)}^{\sum_{j=1}^{m_{i}}\left(d_{ij}r_{ij}+ts_{ij}r_{ij}^{\prime }\right)}\cdot e{\left(h,h\right)}^{\sum_{j=1}^{m_{i}}r_{ij}r_{ij}^{\prime }} \end{array}$$

Finally, when $ph_{i}$ drives within the transmission range of an RSU, it submits $\phi_{i}$ together with all pseudo-IDs in $V_{i}$ to CS via RSU.

Global Aggregation: Upon receiving reports from all PH vehicles in P, CS aggregates those data together as follows, and passes the final result Φ and all pseudo-IDs of PM vehicles to SP.

(6) $$\begin{array}{cc} & \Phi =\prod_{i=1}^{m}\phi_{i}=e{\left(g,g\right)}^{\sum_{i=1}^{m}\sum_{j=1}^{n_{i}}d_{ij}\cdot ts_{ij}}\\ & \cdot e{\left(g,h\right)}^{\sum_{i=1}^{m}\sum_{j=1}^{m_{i}}\left(d_{ij}r_{ij}+ts_{ij}r_{ij}^{\prime }\right)}\cdot e{\left(h,h\right)}^{\sum_{i=1}^{m}\sum_{j=1}^{m_{i}}r_{ij}r_{ij}^{\prime }} \end{array}$$

### 4.4. Aggregated Sensed Data Retrieval

Once SP receives the aggregated sensed data Φ from CS, it retrieves it using its secret key *p*:

(7) $$\begin{array}{cc} & \Phi^{p}=e{\left(g,g\right)}^{p\cdot \sum_{i=1}^{m}\sum_{j=1}^{n_{i}}d_{ij}\cdot ts_{ij}}\\ & \cdot e{\left(g,h\right)}^{p\cdot \sum_{i=1}^{m}\sum_{j=1}^{m_{i}}\left(d_{ij}r_{ij}+ts_{ij}r_{ij}^{\prime }\right)}\cdot e{\left(h,h\right)}^{p\cdot \sum_{i=1}^{m}\sum_{j=1}^{m_{i}}r_{ij}r_{ij}^{\prime }}\\ & =e{\left(g,g\right)}^{p\cdot \sum_{i=1}^{m}\sum_{j=1}^{n_{i}}d_{ij}\cdot ts_{ij}} \end{array}$$

Similarly, we have:

(8) $$\begin{array}{cc} & e{\left(h,h\right)}^{p\cdot \sum_{i=1}^{m}\sum_{j=1}^{m_{i}}r_{ij}r_{ij}^{\prime }}=e{\left(h,h^{p}\right)}^{\sum_{i=1}^{m}\sum_{j=1}^{m_{i}}r_{ij}r_{ij}^{\prime }}\\ & =e{\left(h,1\right)}^{\sum_{i=1}^{m}\sum_{j=1}^{m_{i}}r_{ij}r_{ij}^{\prime }}=1 \end{array}$$

Since the aggregated data $\sum_{i=1}^{m}\sum_{j=1}^{n_{i}}d_{ij}\cdot ts_{ij}$ is in a small space, we can use the method of exhaustion to retrieve them. From the pseudo-IDs in $V_{i},i\in \left[1,m\right]$, SP is able to find their real identities and corresponding trust scores $ts_{ij},i\in \left[1,m\right],j\in \left[1,n_{i}\right]$. Then, SP computes the sensed data $d_{0}$ using a weighted majority method:

(9) $$d_{0}=\frac{\sum_{i=1}^{m}\sum_{j=1}^{n_{i}}d_{ij}\cdot ts_{ij}}{\sum_{i=1}^{m}\sum_{j=1}^{n_{i}}ts_{ij}}$$

For each category, there will be a sensed result; therefore the sensed result vector $\vec{d_{0}}$ will be $\vec{d_{0}}=\left(d_{0}^{A},d_{0}^{B},\cdots ,d_{0}^{Z}\right)$. After shrinking 100 times, SP will get the final sensed result.

### 4.5. Privacy-Preserving Sensed Data Accuracy Evaluation

We assume that all PM vehicles in each PH vehicle $ph_{i}$’s platoon contribute their sensed data in the task, where $ph_{i}\in P,i\in \left[1,m\right]$. These PM vehicles form a set, denoted as $V=\{v_{1},v_{2},\cdots ,v_{n}\}$, where *n* is the total number of these PM vehicles. After the sensed result is computed, SP would like to evaluate the sensed data accuracy in this task for each PM vehicle who contributes its data. Specifically, for $v_{k}\in V$, from Section 4.3, we learn that CS stores $v_{k}$’s pseudo-ID and encrypted sensed data for sensing category *A* as: $D_{k}^{A}=g^{d_{k}^{A}}\cdot h^{{r^{\prime }}_{k}^{A}}$. The evaluation score $f_{k}\in \left[0,1\right]$ for $v_{k}$ in this task can be calculated by following the steps below:

*Step 1:* Given that there are many sensing categories in the sensed data vector, SP first defines a tolerance value for each sensing category in sensed result vector $\vec{d}$. We denote these tolerance values as another vector $\vec{d_{t}}=\left(d_{t}^{A},d_{t}^{B},\cdots ,d_{t}^{Z}\right)$. The tolerance value can be explained in this way: if the difference between the sensed data and sensed result is larger than tolerance level, the sensed data accuracy is unacceptable and $f_{k}=0$. In addition, SP also defines the weights for different sensing categories as $\omega_{A},\omega_{B},\cdots$ which satisfies $\omega_{A}+\omega_{B}+\cdots +\omega_{Z}=1$.

*Step 2:* We take sensing category *A* as an example, and SP needs to calculate the difference between sensed result and sensed data $\Delta d_{k}^{A}=\left|d_{0}^{A}-d_{k}^{A}\right|$. When there are many vehicles in the VANET, the computation costs are to large for SP so it should be done by CS in a privacy-preserving way as follows. Note that, in case $d_{0}^{A}$ is not an integer, SP rounds it off to the nearest integer.

- SP encrypts the sensed result $d_{0}^{A}$ as: $D_{0}^{A}=g^{d_{0}^{A}}\cdot h^{{r^{\prime }}_{0}^{A}}$, where ${r^{\prime }}_{0}^{A}\in Z_{n}^{*}$, and sends $D_{0}^{A}$ to CS.
- CS pairs the encrypted sensed data $D_{k}^{A}$ from $v_{k}$ and the inverse of encrypted sensed result, ${D_{k}^{A}}^{-1}$, as: $\alpha_{k}^{A}=e\left(D_{0}^{A},{D_{k}^{A}}^{-1}\right)$. In the case that $d_{0}<d_{k}$, CS also pairs the data as: $\beta_{k}^{A}=e\left({D_{0}^{A}}^{-1},D_{k}^{A}\right)$. After calculation, CS passes the results together with the $v_{k}$’s pseudo-ID $\left\{PID_{k}||\alpha_{k}^{A}||\beta_{k}^{A}\right\}$ to SP.
- Upon receiving CS’s message, SP first finds the real identity of $v_{k}$ according to its pseudo-ID $PID_{k}$, then retrieves $\Delta d_{k}^{A}=\left|d_{0}^{A}-d_{k}^{A}\right|$ using the same method of exhaustion proposed in Section 4.4.

*Step 3:* After calculating $\Delta d_{k}^{A}$, SP uses a similar way to calculate other sensing categories as: $\Delta d_{k}^{B},\Delta d_{k}^{C},\cdots ,\Delta d_{k}^{Z}$. Finally, the evaluation score for $v_{k}$ in this task is calculated by:

(10) $$f_{k}=\omega_{A}\left(1-\frac{\Delta d_{k}^{A}}{d_{t}^{A}}\right)+\omega_{B}\left(1-\frac{\Delta d_{k}^{B}}{d_{t}^{B}}\right)+\cdots +\omega_{Z}\left(1-\frac{\Delta d_{k}^{Z}}{d_{t}^{Z}}\right)$$

### 4.6. Dirichlet-Based Trust Management

For a specific PM vehicle $v_{k}\in V$, the SP would like to evaluate its trustworthiness from its evaluation scores. Since the trustworthiness of $v_{k}$ reflects its performance in a long period, SP first collects $v_{k}$’s evaluation scores in many tasks, denoted by a continuous random variable *X* (0≤X≤1). From these collected historic records, SP can estimate *X*’s future distributions by using Dirichlet distribution. Since Dirichlet distribution is based on initial belief on an unknown event according to prior distribution, it provides a solid mathematical foundation for measuring the uncertainty of feedbacks based on historical data. Compared to Beta distribution, which is more appropriate in a binary satisfaction level [15], Dirichlet distribution is more appropriate for multi-valued satisfaction levels [16]. In our case, the evaluation trustworthiness of user vehicles are described by continuous trust scores. Therefore, SP uses Dirichlet distribution to estimate the performance of candidate vehicles in the future and then builds the trust model accordingly.

In order to classify the historical and future evaluation scores, we also denote a number of *l* satisfaction levels of feedback as a set $\left\{\theta_{1},\theta_{2},\cdots ,\theta_{l}\right\}$ $\left(\theta_{i}\in \left(0,1\right],i\in \left[1,l\right],\theta_{i}<\theta_{i+1}\right)$. Let $\vec{p}=\left\{p_{1},p_{2},\cdots ,p_{l}\right\}\left(\sum_{i=1}^{l}p_{i}=1\right)$ be the probability distribution vector of *X* with respect to satisfaction levels, so that we have $P\left\{\theta_{i-1}<X_{i}\leq \theta_{i}\right\}=p_{i}\left(i=1,2,\cdots ,l\right)$. To make it more mathematically precise, we define $\theta_{0}=0$ when i=1, $X_{i}=0$ is categorized into $\theta_{1}$.

As described in Section 4.5, once $v_{k}$ finishes many sensing tasks, the SP is able to collect $v_{k}$’s historical evaluation scores. Then, we let $\vec{\gamma }=\left\{\gamma_{1},\gamma_{2},\cdots ,\gamma_{l}\right\}$ denote the vector of cumulative evaluation score and initial belief of *X*. With a posterior Dirichlet distribution, $\vec{p}$ can be modeled as:

(11) $$f\left(\vec{p}|\xi \right)=Dir\left(\vec{p}|\vec{\gamma }\right)=\frac{\Gamma (\Sigma_{i=1}^{l}\gamma_{i})}{\prod_{i=1}^{l}\Gamma \left(\gamma_{i}\right)}\prod_{i=1}^{l}p_{i}^{\gamma_{i}-1}$$

where *ξ* denotes the background information represented by $\vec{\gamma }$. Let: $\gamma_{0}=\sum_{i=1}^{l}\gamma_{i}$. The expected value of the probability of $X_{i}\in \left(\theta_{i-1},\theta_{i}\right]$ with the historical distribution of evaluation scores is given by:

(12) $$E\left(p_{i}|\vec{\gamma }\right)=\frac{\gamma_{i}}{\gamma_{0}}$$

Consider the time factor of historical evaluation scores, and we introduce a forgetting factor *η* and give greater weight to more recent evaluation scores:

(13) $$\vec{\gamma^{\left(n\right)}}=\left\{\begin{array}{c} {\vec{S}}^{\left(0\right)}\;\;\;\;\;\;\;\;\;\;\;\;\;\;\;\;\;\;\;\;\;\;\;\;\;\;\;\;\;\;\;\;\;\;\left(n=0\right)\\ \sum_{i=1}^{n}\eta^{t-t_{i}}{\vec{S}}^{\left(i\right)}+c_{0}{\vec{S}}^{\left(0\right)}\;\;\;\;\;\;\;\;\;\left(n\geq 1\right) \end{array}\right.$$

where *n* is the total number of historical evaluation scores, and $\vec{S^{\left(0\right)}}$ is the initial belief vector when n=0. Since no prior information is available, all elements of $\vec{S^{\left(0\right)}}$ have equal probability, which makes $\vec{S^{\left(0\right)}}=\left(\frac{1}{l},\frac{1}{l},\cdots ,\frac{1}{l}\right)$. Parameter $c_{0}>0$ is a weight on the initial beliefs. In the *i*th sensing task of $v_{k}\left(i\in \left[1,n\right]\right)$, $\vec{S^{\left(i\right)}}$ denotes the satisfaction level of its evaluation score, which contains only one element set to 1, corresponding to the selected satisfaction level, and all the other l−1 elements set to 0. $t_{i}$ stands for the timestamp when the *i*th task took place and *t* is the moment of running the algorithm. The forgetting factor is η∈[0,1], and a smaller *η* means that it is easier for the system to forget the historical records and *vice versa*. In order to defend against on-off attack [17], we choose an adaptive value as: $\eta =c_{1}\cdot \left(1-ts_{k}\right)$, where $c_{1}$ is a parameter to control the forgetting factor, and the larger value of $c_{1}$ makes the system more forgettable about the historical behaviors and *vice versa*. From the equation we can see that when $v_{k}$ has a high trust score, its forgetting factor is small, which means that those good performances will be easily forgotten. On the contrary, once $v_{k}$ provides low accuracy sensed data, its trust score gets lower and the forgetting factor becomes larger. This means that all of those poor sensing performances will be memorized, and it takes even longer time for $v_{k}$ to build up a high trust score again.

To calculate $v_{k}$’s trust score when a sensing vehicle, we first assign the weight $\omega_{i}$ to each satisfaction level $\theta_{i}\left(i\in \left[1,l\right]\right)$. Let $p_{i}$ denote the probability that $v_{k}$’s evaluation score is categorized into the satisfaction level of $\theta_{i}$. $\vec{p}=\left(p_{1},p_{2},\cdots ,p_{l}\right)|\sum_{i=1}^{l}p_{i}=1$. We model $\vec{p}$ using Equations (11)–(13). Let *Y* be the random variable denoting the weighted average of the probability of each evaluation score in $\vec{p}$, and the trust score $ts_{k}$ of $v_{k}$ is represented as:

(14) $$ts_{k}=E\left[Y\right]=\sum_{i=1}^{l}\omega_{i}E\left[p_{i}\right]=\frac{1}{\gamma_{0}}\sum_{i=1}^{l}\omega_{i},\gamma_{i}$$

where $\gamma_{i}$ is the accumulated evidence that $v_{k}$’s evaluation score is with a satisfaction level of $\theta_{i}$.

## 5. Security Analysis

In this section, we discuss the security and privacy properties of the proposed TripSense scheme. In particular, following the design goals discussed early, we examine whether the proposed TripSense scheme can achieve the desirable security and privacy requirements.

### 5.1. The Proposed TripSense Scheme Is Privacy-Preserving for PM Vehicles

- *PM vehicle’s identity privacy and location privacy are preserved in the proposed TripSense scheme:* In our proposed TripSense scheme, each PM vehicle $v_{j}\in V_{0}$ uses pseudo-ID $PID_{jk}$ instead of a real identity in the network. Hence, the identity privacy can be achieved. In addition, to preserve the location privacy of the PM vehicle, $v_{j}$ changes its unlinkable pseudo-IDs at different trips and locations to ensure that its past and future trip and location information will not be linked by pseudo-IDs.
  However, as analyzed in Section 2.2, $v_{j}$ still suffers from trust score link attack. Thus, in our proposed scheme, when a PH vehicle $ph_{i}$ checks whether its PM vehicle $v_{j}$’s trust score satisfies the task requirement, it uses discrete trust levels $TL_{x}$ in place of accurate trust scores. In other words, $v_{j}$ can prove itself a highly trusted vehicle in front of $ph_{i}$ without revealing its exact trust score. In addition, $v_{j}$’s trust score $ts_{j}$ is encrypted as $C=g^{ts_{j}}h^{r}$ and its trust level is in PS’s signature $A_{jk}=g^{\frac{1}{y_{x}+ts_{j}+s_{jk}+H\left(T_{j}\right)}}$, which makes it impossible for the other PM vehicles to get either $v_{j}$’s trust score or trust level.
- *The sensed data privacy preservation is achieved:* Once the sensed data are aggregated by a PM vehicle $v_{j}$, they are encrypted as: $D=g^{d_{j}}\cdot h^{r^{\prime }}$. In the whole process of local aggregation and global aggregation, those data are all aggregated without decryption until SP is reached, where SP is able to recover with its private key *p*. Therefore, unless the other vehicles know the private key *p*, the sensed data information will never be disclosed.

### 5.2. The Proposed TripSense Scheme Achieves Robustness Against Attacks Launched by Adversary

- *Resilience to malicious sensing attack:* According to our proposed scheme, the selfish or malicious vehicles that submit arbitrary sensed data will get low evaluation scores in the trust system. Those low evaluation scores will be accumulated and finally lead to low trust scores if they keep behaving in that way. When their trust scores are lower than threshold $TL_{TH}$, their sensed data will be excluded from data aggregation or they will be given a low weight in data aggregation due to low trust scores. In both ways, the attacker will be mitigated in our proposed scheme.
- *Resilience to Trust Score Spoofing Attack:* We assume that the majority of PM vehicles follow the scheme honestly, but we do not rule out a possibility that a small fraction of PM vehicles cheat PH vehicles by using the fake trust scores. There are two possible cases: one case is that the PM vehicle $v_{j}$ spoofs a higher trust score $ts_{j}^{\prime }$ with the hope to participate in a sensing task. However, in this case, when $v_{j}$ is using pseudo-ID $PID_{jk}$, $v_{j}$’s trust score $ts_{j}$ is signed by SP as $A_{jk}=g^{\frac{1}{y_{x}+ts_{j}+s_{jk}+H\left(T_{j}\right)}}$, where $y_{x}$, $s_{jk}$, $H(T_{j})$ indicate the trust level, private key and updating timestamp, respectively. Therefore, without knowing the spoofed trust score $ts_{j}^{\prime }$’s master key $y_{x}^{\prime }$, $v_{j}$ is unable to launch attack. Another case is that $v_{j}$ provides a fake trust score $ts_{j}^{\prime }$ after encryption as $C^{\prime }=g^{ts_{j}^{\prime }}h^{r^{\prime }}$, $E^{\prime }=B^{ts_{j}^{\prime }}g^{v}$. To deal with this type of attack, PH vehicle needs to check $\phi \overset{?}{=}H\left(C,B,E,D,\hat{C},\hat{E},H{\left(T\right)}^{s_{i}}\right)$, it won’t hold once the original *C* and *E* are changed to $C^{\prime }$ and $E^{\prime }$.
- *Resilience to Impersonation Attack:* Both PH vehicle and PM vehicle could be impersonated by unqualified vehicles that want to take part in the sensing tasks. Specifically, for an impersonated PM vehicle, it may submit false data and escape punishments; for a PH vehicle, it may collect sensed data without submitting to CS and render the sensed data results incomplete. However, this attack can be thwarted by our proposed scheme. In the initialization phase in Section 4.1, both PH and PM vehicles will be given a pair of private and public keys once they are registered. In Section 4.2, a PM vehicle $v_{j}$ first checks PH vehicle $ph_{i}$ by checking $e\left(H{\left(T\right)}^{s_{i}},g\right)\overset{?}{=}e\left(S_{i},H\left(T\right)\right)$ before it accepts $ph_{i}$’s sensing task requirement. The timestamp H(T) is used to resist against replay attack. Similarly, the PH vehicle $ph_{i}$ also checks $e\left(g,H{\left(T^{\prime }\right)}^{s_{jk}}\right)\overset{?}{=}e\left(S_{jk},H\left(T^{\prime }\right)\right)$ before accepting the sensed data from $v_{j}$. As a result, our proposed TripSense scheme is resistant against impersonation attacks.

## 6. Performance Evaluation

We will evaluate the performance of our proposed TripSense scheme in this section, the numerical data is generated in MATLAB. The performance metrics used in the evaluation are: (i) *trust score variations* for different PM vehicles in terms of the task number; (ii) *detection ratio* defined as the ratio of the number of detected malicious vehicles with respect to the total number of malicious vehicles with the increase of task number.

### 6.1. Simulation Settings

We design a simulation to evaluate our proposed TripSense scheme in which only a set of key factors are considered and specified in order to validate the PM vehicles’ sensing accuracy. It is worth noting that the selected factors are related to the movement of vehicles and the packet collision problems. In this case, we simulate the proposed scheme in the environment of MATLAB where there are a total number of *n* registered PM vehicles. To ensure the fairness, we suggest that each PM vehicle provides *m* times sensing report in totally *m* independent tasks. The detailed simulation parameter settings is in Table 1.

### 6.2. Modeling the Sensing Behaviors of PM Vehicles

Due to the lack of real data, we need to model the behaviors of not only PM vehicles who take part in the sensing tasks, especially for malicious vehicles, in order to test the performance of our proposed scheme.

**Sensing accuracy level (SAL) of PM vehicles:** We define a parameter as sensing accuracy level (PQL) $l_{v}\in \left[0,1\right]$ to describe the capability of a PM vehicle to provide high accuracy sensed data. A PM vehicle with higher $l_{v}$ may submit more accurate sensing reports. Specifically, given a PM vehicle with $l_{v}$, we use the beta distribution to describe the performance quality variable *X* of that PM vehicle, the probability density function of beta distribution can be expressed as:

(15) $$f\left(x|\alpha ,\beta \right)=\frac{\Gamma (\alpha +\beta )}{\Gamma (\alpha )\Gamma (\beta )}x^{\alpha -1}{\left(1-x\right)}^{\beta -1}$$

where $\Gamma \left(\alpha \right)=\int_{0}^{\infty }x^{\alpha -1}e^{-x}dx$. f(x|α,β) is the probability that a PH vehicle with PQL of $l_{v}$ provides a service with the quality value of x∈[0,1]. Higher values of $l_{v}$ imply that the PH vehicle provides a higher quality service. To achieve this goal, we define *α* and *β* as follows:

(16) $$\begin{array}{cc} & \alpha =c_{2}\cdot l_{v}\\ & \beta =c_{2}\cdot \left(1-l_{v}\right) \end{array}$$

where $c_{2}$ is the parameter to control the variance of the distribution. When $c_{2}$ is given a larger value, the performance quality values will have a larger variance and *vice versa*. For a PH vehicle with SAL of $l_{v}$, the above model has the property of generating a service quality score that follows a beta distribution with the expectation $E\left(X\right)=l_{v}$. We define that the malicious vehicles are vehicles with SAL $l_{v}\leq 0.2$.

### 6.3. Simulation Results

#### 6.3.1. Correctness

**Figure 4:**
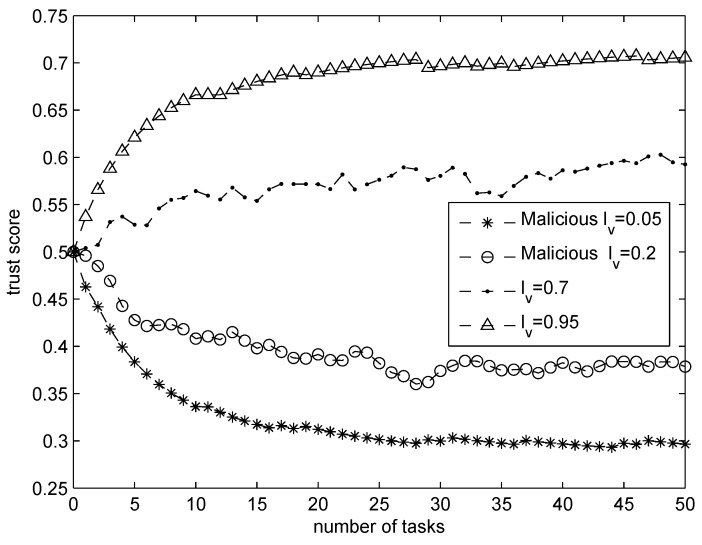
Trust score comparison between honest platoon member vehicles with $l_{v}=0.7$, $l_{v}=0.95$ and malicious PM vehicles with $l_{v}=0.05$, $l_{v}=0.2$.

In this experiment, we target comparing the trust scores between malicious and honest sensing PM vehicles with different sensing accuracy levels (SALs). For a better comparison, we choose two honest PM vehicles with FAL of $l_{v}=0.7$ and $l_{v}=0.95$, respectively. In addition, other malicious PM vehicles who provide corrupt sensed data are also put into the system. After “50” number of tasks, we plot their trust scores in Figure 4.

We notice that the trust scores of all PM vehicles converge after “30” tasks. It is obvious that the honest PM vehicles with $l_{v}=0.7$ and $l_{v}=0.95$ get the highest trust scores after the experiments. On the contrary, both of the attackers get the low trust scores. We also notice that a PM vehicle with larger SAL will achieve higher trust scores, which shows the correctness of our trust model to identify PM vehicles according to their actual SALs.

#### 6.3.2. Effectiveness

To demonstrate the effectiveness of our proposed scheme in detecting malicious PM vehicles, we define a proportion of ρ=20% number of PM vehicles with the lowest $l_{v}$ as “malicious PM vehicles”. After the m=50 tasks, all PM vehicles will be re-ranked, so the detection ratio is defined as the ratio of “malicious PM vehicles” who remain lowest 20% in the new ranking list.

**Figure 5:**
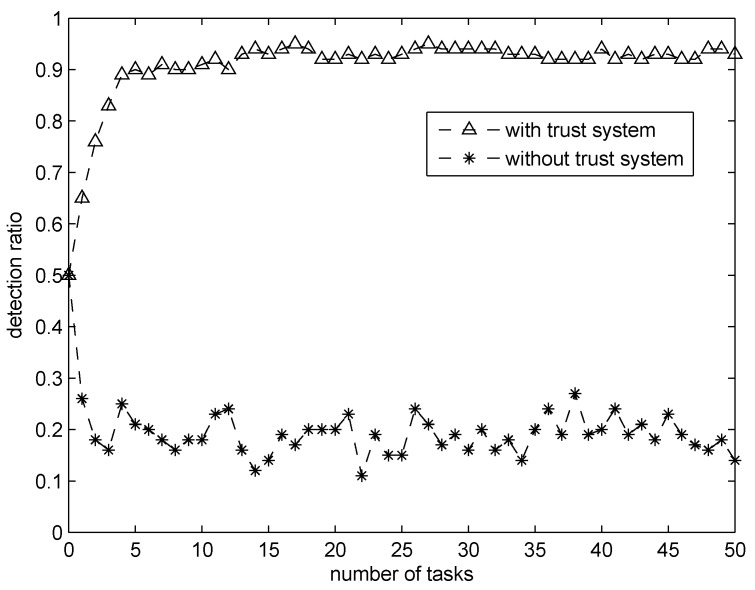
Detection ratio comparison between sensing system with/without trust system.

Figure 5 depicts the detection ratio between our proposed trust-based sensing system with a sensing system without trust. From the figure, we can see that our proposed system’s detection ratio increases quickly with the increase of task numbers, and, after around 5 tasks, it will be convergent to 92%. On the contrary, for a sensing system without a trust system, the selection of sensing PM vehicle is random and the detection ratio remains as low as 20%. Therefore, the effectiveness of our proposed scheme has been demonstrated.

## 7. Related Work

Recently, a lot of research has appeared on trust and reputation management in VANET [18,19,20,21], privacy preserving data aggregation [22,23,24] and crowdsensing [5,25], which are closely related to the techniques in our proposed TripSense scheme.

For trust and reputation management, Zhang *et al.* have done a survey for effective trust management in VANET in [18]. Specifically, it discusses challenging issues for trust management caused by the important characteristics of VANET environments, and points out that robustness should receive particular attention. Patwardhan *et al.* present a distributed reputation management scheme for VANET, which enables vehicles to quickly adapt to changing local conditions and provides a bootstrapping method for establishing trust relationships [19]. However, their scheme is not quite scalable and robust. Different from the traditional entity-based trust model, Raya *et al.* suggest a data-oriented trust establishment framework [20]. By combining trust values of each piece of data together, their framework deals well with ephemerality and functions well in sparse areas. However, in dense urban areas, due to large amounts of data, their framework is less efficient.

There has also been extensive work on data aggregation schemes in VANET [26,27]. These works share the same assumption that vehicles or servers are trusted and the communications are secure, which, however, is not the case in real scenarios. In reality, data can be eavesdropped on and disclosed. Therefore, a lot of work has been done in privacy-preservation data aggregation [22,23,24]. Xing *et al.* have proposed M-PERM, a mutual privacy-preserving regression modeling approach to address the issue of keeping both participants and user data private while still utilizing them for analysis [22]. In this paper, data are aggregated at each node and each cluster, and finally at the user with maximum privacy protection. He *et al.* present two privacy-preserving data aggregation schemes for additive aggregation functions, which bridge the gap between collaborative data collection and data privacy [23]. Bilogrevic *et al.* have proposed a state-of-the-art privacy preservation framework to preserve data utility and simultaneously provide user privacy [24]. Users in this framework only contribute encrypted and aggregated models of their files to the aggregator to tackle trust and incentive challenges.

Burke is the first to introduce the concept of participatory sensing, and describes an initial architecture to enhance data credibility, quality, privacy, and `shareability’ [25]. Ganti gives an overview of crowdsensing by introducing existing mobile crowdsensing applications and explaining their unique characteristics, illustrating various research challenges, and discussing possible solutions [5].

Combining the above privacy preserving data aggregation techniques and trust models together, our proposed TripSense scheme is focused on evaluating the platoon member vehicles’ sensing ability based on the accuracy of their historical sensed data. Specifically, there are several aspects which make our proposed scheme different: first, we establish a trust system as a long-term evaluating metric. Second, we make use of platoon head vehicles for authentication of local data aggregation, which greatly reduces the communication overhead between vehicles and infrastructures, hence making it very suitable for VANET. Third, our proposed scheme is privacy-preserving on platoon member vehicles’ identities, locations and data.

## 8. Conclusions

In this paper, we have proposed a trust-based privacy-preserving scheme for vehicular platoon crowdsensing called TripSense. The proposed scheme mainly focuses on establishing a trust model to improve the sensed data reliability and accuracy of the whole system, while preserving the location and data privacy of sensing vehicles in the process of sensing vehicle selection, sensed data aggregation and evaluation. Detailed security analysis shows that the proposed TripSense scheme can not only achieve vehicle’s identity privacy, location privacy and data privacy, but it also is resistant against adversary attacks on malicious sensing reports. Moreover, through extensive performance evaluation, we have demonstrated that our proposed scheme can achieve better sensing accuracy. In our future work, we will consider more scenarios in crowdsensing rather than data aggregation. In addition, we may also consider the collusion among PM and PH vehicles to launch attacks in order to victimize other vehicles.

## Figures and Tables

**Table 1:** Simulation Parameter Settings.

Notation	Definition	Value
*n*	registered PM vehicle number	500
*ρ*	malicious PH vehicle proportion	20%
$l_{v}$	sensing accuracy level (SAL)	0.05; 0.2; 0.7; 0.95
*m*	number of tasks for each PM vehicle	50
$c_{0}$	initial belief weight	1
$c_{1}$	forgetting factor parameter	1
$c_{2}$	variance sensitivity	10
$c_{3}$	forgetting factor parameter	1
$T_{0}$	initial trust score	0.5
